# Methyl 3,5-bis­[(3-chloro­pyrazin-2-yl)­oxy]benzoate

**DOI:** 10.1107/S1600536813010465

**Published:** 2013-04-20

**Authors:** Thothadri Srinivasan, Venkatesan Kalpana, Perumal Rajakumar, Devadasan Velmurugan

**Affiliations:** a Centre of Advanced Study in Crystallography and Biophysics, University of Madras, Guindy Campus, Chennai 600 025, India; b Department of Organic Chemistry, University of Madras, Guindy Campus, Chennai 600 025, India

## Abstract

In the title compound, C_16_H_10_Cl_2_N_4_O_4_, the pyrazine rings make dihedral angles of 67.82 (9) and 75.91 (9)° with the benzene ring, while the dihedral angle between the pyrazine rings is 44.69 (10)°. The meth­oxy­carbonyl group makes a dihedral angle of 16.82 (8)° with the benzene ring to which it is attached. In the crystal, C—H⋯O hydrogen bonds link the mol­ecules, forming chains running along the *ab* plane.

## Related literature

For applications of the pyrazine ring system in drug development, see: Du *et al.* (2009 ▶); Dubinina *et al.* (2006 ▶); Ellsworth *et al.* (2007 ▶); Mukaiyama *et al.* (2007 ▶). For a related structure, see: Nasir *et al.* (2010 ▶).
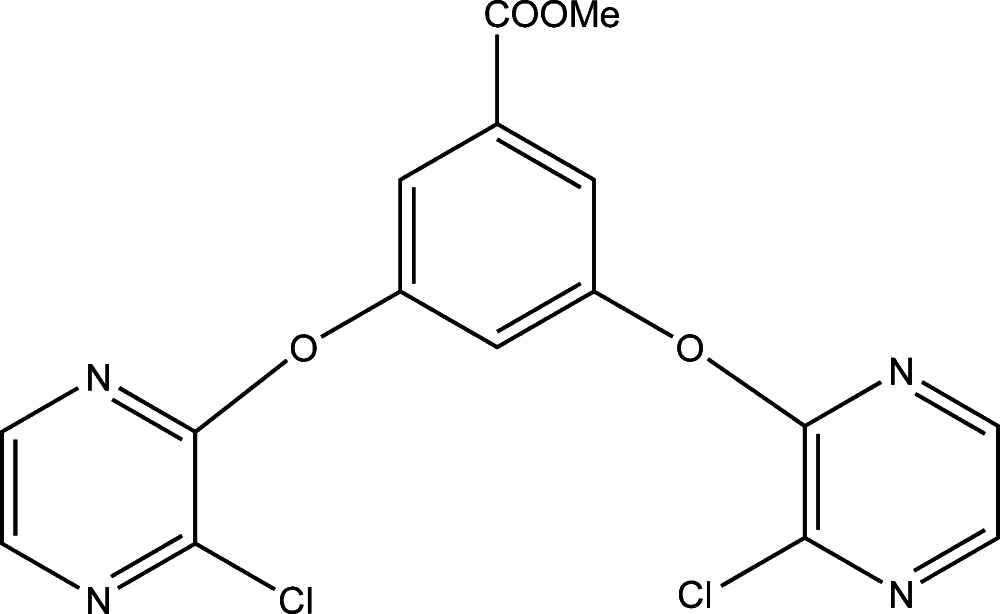


## Experimental

### 

#### Crystal data

C_16_H_10_Cl_2_N_4_O_4_
*M* *_r_* = 393.18
Triclinic, 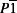

*a* = 8.5437 (15) Å
*b* = 9.2984 (18) Å
*c* = 11.545 (2) Å
α = 88.592 (10)°
β = 74.974 (9)°
γ = 73.231 (9)°
*V* = 846.9 (3) Å^3^
*Z* = 2
Mo *K*α radiation
μ = 0.41 mm^−1^
*T* = 293 K
0.30 × 0.25 × 0.20 mm

#### Data collection

Bruker SMART APEXII area-detector diffractometer
Absorption correction: multi-scan (*SADABS*; Bruker, 2008 ▶) *T*_min_ = 0.886, *T*_max_ = 0.922
12425 measured reflections
3453 independent reflections
3076 reflections with *I* > 2σ(*I*)
*R*_int_ = 0.024

#### Refinement

*R*[*F* ^2^ > 2σ(*F* ^2^)] = 0.039
*wR*(*F* ^2^) = 0.124
*S* = 1.04
3453 reflections
236 parameters
H-atom parameters constrained
Δρ_max_ = 0.30 e Å^−3^
Δρ_min_ = −0.46 e Å^−3^

### 

Data collection: *APEX2* (Bruker, 2008 ▶); cell refinement: *SAINT* (Bruker, 2008 ▶); data reduction: *SAINT*; program(s) used to solve structure: *SHELXS97* (Sheldrick, 2008 ▶); program(s) used to refine structure: *SHELXL97* (Sheldrick, 2008 ▶); molecular graphics: *ORTEP-3 for Windows* (Farrugia, 2012 ▶); software used to prepare material for publication: *SHELXL97* and *PLATON* (Spek, 2009 ▶).

## Supplementary Material

Crystal structure: contains datablock(s) global, I. DOI: 10.1107/S1600536813010465/pv2625sup1.cif

Structure factors: contains datablock(s) I. DOI: 10.1107/S1600536813010465/pv2625Isup2.hkl

Supplementary material file. DOI: 10.1107/S1600536813010465/pv2625Isup3.cml

Additional supplementary materials:  crystallographic information; 3D view; checkCIF report

## Figures and Tables

**Table 1:** Hydrogen-bond geometry (Å, °)

*D*—H⋯*A*	*D*—H	H⋯*A*	*D*⋯*A*	*D*—H⋯*A*
C4—H4⋯O2^i^	0.93	2.47	3.192 (3)	135

Symmetry code: (i) 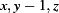.

**Fig. 1.**
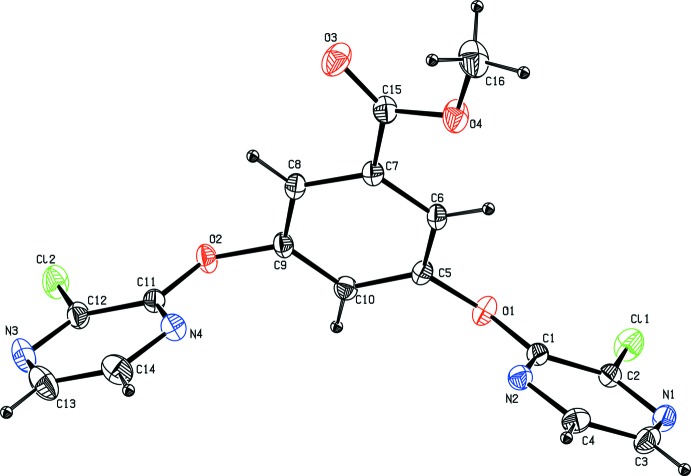
The molecular structure of the title compound, showing displacement ellipsoids drawn at the 30% probability level. H atoms are presented as small spheres of arbitrary radius.

**Fig. 2.**
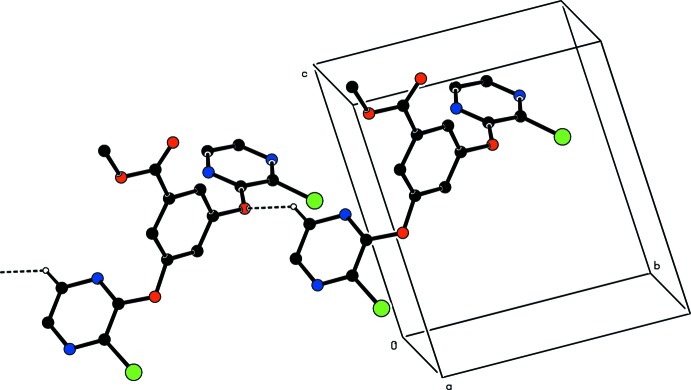
The crystal packing of the title compound viewed down a axis. H-atoms not involved in H-bonds have been excluded for clarity.

## supplementary crystallographic information

### Comment

The pyrazine ring is a useful structural unit in medicinal chemistry and has found broad applications in drug development and can be used as antiproliferative agent (Dubinina *et al.*, 2006), potent CXCR3 antagonist (Du *et al.*, 2009), CB1 antagonist (Ellsworth *et al.*, 2007) and c-Src inhibitor (Mukaiyama *et al.*, 2007). In view of different applications of this class of compounds, we have undertaken the single-crystal structure determination of the title compound.

The bond distances and angles in the title compound (Fig. 1) agree very well with the corresponding bond distances and angles reported in a closely related compound (Nasir *et al.*, 2010). In the title compound, the pyrazine ring (N1/N2/C1-C4) makes a dihedral angle of 67.82 (9)° with the benzene ring (C5-C10). The other pyrazine ring (N3/N4/C11-C14) makes a dihedral angle of 75.91 (9)° with the bezene ring. The dihedral angle between the two pyrazine rings is 44.69 (10)°. Moreover, the acteyl group (C15/03/04/C16) attached with the benzene ring makes a dihedral angle of 16.82 (8)° with the aryl ring. The chlorine atoms Cl1 and Cl2 attached with the pyrazine rings deviate by 0.0136 (6)Å and 0.0590 (7)Å. The crystal packing is stabilised by intermolecular C4–H4···O2 hydrogen bonds (Tab. 1 & Fig. 2).

### Experimental

To a stirred solution of Cs_2_CO_3_/K_2_CO_3_ (22 mmol) in CH_3_CN (50 mL), methyl-3,5-dihydroxybenzoate (10 mmol) was added and stirred for 5 min. 2,3-Dichloropyrazine (20 mmol) in CH_3_CN (100 mL) was added dropwise to the above reaction mixture and allowed for stirring at refluxing condition for 12 h. After the reaction was complete, the reaction mixture was allowed to attain room temperature and then evaporated to dryness. The residue obtained was extracted with CH_2_Cl_2_ (3 x 100 mL), washed with water (3 x 100 mL), brine and then dried over Na_2_SO_4_. Evaporation of the organic layer gave a residue, which on purification using column chromatography with hexane/CHCl_3_ (1:1) as an eluent gave the title compound. Single crystals suitable for X-ray diffraction were obtained by slow evaporation of a solution of the title compound in hexane at room temperature.

### Refinement

The hydrogen atoms were placed in calculated positions with C—H = 0.93 and 0.96 Å, for aryl and methyl type H-atoms, respectively, and refined in the riding model with fixed isotropic displacement parameters: *U*_iso_(H) = 1.5*U*_eq_(C) for methyl and *U*_iso_(H) = 1.2*U*_eq_(C) for aryl H-atoms.

### Crystal data

**Table:**

C_16_H_10_Cl_2_N_4_O_4_	*Z* = 2
*M**_r_* = 393.18	*F*(000) = 400
Triclinic, *P*1	*D*_x_ = 1.542 Mg m^−^^3^
Hall symbol: -P 1	Mo *K*α radiation, λ = 0.71073 Å
*a* = 8.5437 (15) Å	Cell parameters from 3453 reflections
*b* = 9.2984 (18) Å	θ = 1.8–26.6°
*c* = 11.545 (2) Å	µ = 0.41 mm^−^^1^
α = 88.592 (10)°	*T* = 293 K
β = 74.974 (9)°	Block, colourless
γ = 73.231 (9)°	0.30 × 0.25 × 0.20 mm
*V* = 846.9 (3) Å^3^	

### Data collection

**Table:**

Bruker SMART APEXII area-detector diffractometer	3453 independent reflections
Radiation source: fine-focus sealed tube	3076 reflections with *I* > 2σ(*I*)
Graphite monochromator	*R*_int_ = 0.024
ω and φ scans	θ_max_ = 26.6°, θ_min_ = 1.8°
Absorption correction: multi-scan (*SADABS*; Bruker, 2008)	*h* = −10→10
*T*_min_ = 0.886, *T*_max_ = 0.922	*k* = −11→11
12425 measured reflections	*l* = −14→14

### Refinement

**Table:**

Refinement on *F*^2^	Primary atom site location: structure-invariant direct methods
Least-squares matrix: full	Secondary atom site location: difference Fourier map
*R*[*F*^2^ > 2σ(*F*^2^)] = 0.039	Hydrogen site location: inferred from neighbouring sites
*wR*(*F*^2^) = 0.124	H-atom parameters constrained
*S* = 1.04	*w* = 1/[σ^2^(*F*_o_^2^) + (0.0678*P*)^2^ + 0.3652*P*] where *P* = (*F*_o_^2^ + 2*F*_c_^2^)/3
3453 reflections	(Δ/σ)_max_ < 0.001
236 parameters	Δρ_max_ = 0.30 e Å^−^^3^
0 restraints	Δρ_min_ = −0.46 e Å^−^^3^

### Special details

**Table:**

Geometry. All esds (except the esd in the dihedral angle between two l.s. planes) are estimated using the full covariance matrix. The cell esds are taken into account individually in the estimation of esds in distances, angles and torsion angles; correlations between esds in cell parameters are only used when they are defined by crystal symmetry. An approximate (isotropic) treatment of cell esds is used for estimating esds involving l.s. planes.
Refinement. Refinement of F^2^ against ALL reflections. The weighted R-factor wR and goodness of fit S are based on F^2^, conventional R-factors R are based on F, with F set to zero for negative F^2^. The threshold expression of F^2^ > 2sigma(F^2^) is used only for calculating R-factors(gt) etc. and is not relevant to the choice of reflections for refinement. R-factors based on F^2^ are statistically about twice as large as those based on F, and R- factors based on ALL data will be even larger.

### Fractional atomic coordinates and isotropic or equivalent isotropic displacement parameters (Å^2^)

**Table:**

	*x*	*y*	*z*	*U*_iso_*/*U*_eq_	
C1	1.2164 (2)	−0.13661 (19)	0.56898 (15)	0.0357 (4)	
C2	1.3326 (2)	−0.2435 (2)	0.48010 (16)	0.0392 (4)	
C3	1.2200 (3)	−0.4217 (2)	0.5674 (2)	0.0512 (5)	
H3	1.2182	−0.5212	0.5714	0.061*	
C4	1.1050 (3)	−0.3171 (2)	0.65286 (19)	0.0486 (4)	
H4	1.0265	−0.3476	0.7125	0.058*	
C5	1.1396 (2)	0.10665 (18)	0.66279 (15)	0.0364 (4)	
C6	1.1808 (2)	0.07829 (19)	0.77108 (16)	0.0377 (4)	
H6	1.2538	−0.0136	0.7823	0.045*	
C7	1.1100 (2)	0.19087 (19)	0.86279 (16)	0.0376 (4)	
C8	0.9977 (2)	0.32842 (19)	0.84678 (16)	0.0394 (4)	
H8	0.9532	0.4046	0.9073	0.047*	
C9	0.9551 (2)	0.34790 (19)	0.73925 (17)	0.0376 (4)	
C10	1.0255 (2)	0.23987 (19)	0.64574 (16)	0.0383 (4)	
H10	0.9970	0.2562	0.5731	0.046*	
C11	0.6780 (2)	0.5146 (2)	0.76707 (15)	0.0376 (4)	
C12	0.5715 (2)	0.6516 (2)	0.74204 (17)	0.0443 (4)	
C13	0.3460 (3)	0.5899 (3)	0.8520 (2)	0.0683 (7)	
H13	0.2294	0.6121	0.8838	0.082*	
C14	0.4482 (3)	0.4569 (3)	0.8760 (2)	0.0594 (6)	
H14	0.3993	0.3908	0.9235	0.071*	
C15	1.1546 (2)	0.1705 (2)	0.98024 (17)	0.0458 (4)	
C16	1.2714 (4)	−0.0079 (3)	1.1083 (2)	0.0749 (8)	
H16A	1.3582	0.0366	1.1128	0.112*	
H16B	1.3125	−0.1150	1.1123	0.112*	
H16C	1.1733	0.0318	1.1742	0.112*	
N1	1.3349 (2)	−0.38465 (18)	0.47831 (15)	0.0479 (4)	
N2	1.10203 (19)	−0.17218 (18)	0.65331 (14)	0.0427 (3)	
N3	0.4081 (2)	0.6891 (2)	0.78443 (18)	0.0618 (5)	
N4	0.6181 (2)	0.41812 (19)	0.83301 (15)	0.0462 (4)	
O1	1.22265 (18)	0.00741 (14)	0.56204 (11)	0.0460 (3)	
O2	0.84699 (15)	0.48570 (14)	0.71782 (13)	0.0445 (3)	
O3	1.1276 (3)	0.27208 (19)	1.05207 (15)	0.0702 (5)	
O4	1.2263 (2)	0.02685 (17)	0.99597 (13)	0.0623 (4)	
Cl1	1.47807 (7)	−0.18931 (7)	0.36871 (5)	0.06146 (19)	
Cl2	0.65455 (7)	0.77453 (6)	0.65015 (6)	0.06183 (19)	

### Atomic displacement parameters (Å^2^)

**Table:**

	*U*^11^	*U*^22^	*U*^33^	*U*^12^	*U*^13^	*U*^23^
C1	0.0370 (8)	0.0322 (8)	0.0376 (8)	−0.0068 (6)	−0.0133 (7)	0.0005 (7)
C2	0.0381 (8)	0.0391 (9)	0.0390 (9)	−0.0076 (7)	−0.0117 (7)	−0.0023 (7)
C3	0.0665 (13)	0.0348 (10)	0.0563 (12)	−0.0176 (9)	−0.0197 (10)	0.0023 (8)
C4	0.0532 (11)	0.0477 (11)	0.0505 (11)	−0.0232 (9)	−0.0137 (9)	0.0039 (9)
C5	0.0381 (8)	0.0292 (8)	0.0387 (9)	−0.0092 (6)	−0.0052 (7)	−0.0010 (7)
C6	0.0351 (8)	0.0289 (8)	0.0434 (9)	−0.0028 (6)	−0.0078 (7)	0.0018 (7)
C7	0.0357 (8)	0.0336 (8)	0.0402 (9)	−0.0070 (7)	−0.0082 (7)	0.0030 (7)
C8	0.0371 (8)	0.0315 (8)	0.0433 (9)	−0.0049 (7)	−0.0050 (7)	−0.0018 (7)
C9	0.0310 (8)	0.0283 (8)	0.0498 (10)	−0.0052 (6)	−0.0087 (7)	0.0068 (7)
C10	0.0391 (9)	0.0361 (9)	0.0410 (9)	−0.0112 (7)	−0.0128 (7)	0.0059 (7)
C11	0.0367 (8)	0.0358 (9)	0.0377 (9)	−0.0049 (7)	−0.0113 (7)	−0.0021 (7)
C12	0.0453 (10)	0.0402 (10)	0.0426 (9)	−0.0009 (8)	−0.0161 (8)	0.0015 (8)
C13	0.0368 (10)	0.0936 (19)	0.0598 (13)	−0.0022 (11)	−0.0063 (9)	0.0058 (13)
C14	0.0465 (11)	0.0787 (16)	0.0515 (12)	−0.0204 (11)	−0.0089 (9)	0.0103 (11)
C15	0.0447 (10)	0.0449 (10)	0.0424 (10)	−0.0061 (8)	−0.0100 (8)	0.0018 (8)
C16	0.0865 (18)	0.0765 (17)	0.0503 (13)	0.0020 (14)	−0.0283 (12)	0.0137 (12)
N1	0.0530 (9)	0.0366 (8)	0.0518 (9)	−0.0062 (7)	−0.0168 (7)	−0.0036 (7)
N2	0.0406 (8)	0.0419 (8)	0.0449 (8)	−0.0126 (6)	−0.0093 (6)	−0.0002 (6)
N3	0.0438 (9)	0.0655 (12)	0.0591 (11)	0.0096 (8)	−0.0132 (8)	0.0027 (9)
N4	0.0418 (8)	0.0467 (9)	0.0483 (9)	−0.0107 (7)	−0.0114 (7)	0.0056 (7)
O1	0.0592 (8)	0.0332 (6)	0.0397 (7)	−0.0130 (6)	−0.0035 (6)	−0.0006 (5)
O2	0.0352 (6)	0.0316 (6)	0.0607 (8)	−0.0030 (5)	−0.0107 (6)	0.0111 (6)
O3	0.1037 (14)	0.0530 (9)	0.0501 (9)	−0.0085 (9)	−0.0293 (9)	−0.0063 (7)
O4	0.0801 (11)	0.0483 (8)	0.0498 (8)	0.0040 (7)	−0.0279 (8)	0.0045 (7)
Cl1	0.0616 (3)	0.0610 (3)	0.0520 (3)	−0.0218 (3)	0.0069 (2)	−0.0066 (2)
Cl2	0.0665 (3)	0.0411 (3)	0.0743 (4)	−0.0066 (2)	−0.0239 (3)	0.0175 (2)

### Geometric parameters (Å, º)

**Table:**

C1—N2	1.300 (2)	C9—C10	1.380 (3)
C1—O1	1.355 (2)	C9—O2	1.4057 (19)
C1—C2	1.407 (2)	C10—H10	0.9300
C2—N1	1.308 (2)	C11—N4	1.294 (2)
C2—Cl1	1.7185 (19)	C11—O2	1.356 (2)
C3—N1	1.340 (3)	C11—C12	1.409 (2)
C3—C4	1.370 (3)	C12—N3	1.297 (3)
C3—H3	0.9300	C12—Cl2	1.718 (2)
C4—N2	1.340 (3)	C13—N3	1.332 (3)
C4—H4	0.9300	C13—C14	1.362 (4)
C5—C6	1.384 (2)	C13—H13	0.9300
C5—C10	1.383 (2)	C14—N4	1.348 (3)
C5—O1	1.398 (2)	C14—H14	0.9300
C6—C7	1.393 (2)	C15—O3	1.203 (3)
C6—H6	0.9300	C15—O4	1.329 (2)
C7—C8	1.401 (2)	C16—O4	1.450 (3)
C7—C15	1.495 (3)	C16—H16A	0.9600
C8—C9	1.375 (3)	C16—H16B	0.9600
C8—H8	0.9300	C16—H16C	0.9600
			
N2—C1—O1	120.32 (16)	C5—C10—H10	120.8
N2—C1—C2	121.66 (16)	N4—C11—O2	120.75 (16)
O1—C1—C2	117.98 (15)	N4—C11—C12	121.81 (17)
N1—C2—C1	122.44 (17)	O2—C11—C12	117.44 (16)
N1—C2—Cl1	117.86 (14)	N3—C12—C11	122.06 (19)
C1—C2—Cl1	119.69 (14)	N3—C12—Cl2	117.28 (15)
N1—C3—C4	121.89 (18)	C11—C12—Cl2	120.64 (15)
N1—C3—H3	119.1	N3—C13—C14	121.9 (2)
C4—C3—H3	119.1	N3—C13—H13	119.0
N2—C4—C3	122.12 (18)	C14—C13—H13	119.0
N2—C4—H4	118.9	N4—C14—C13	122.0 (2)
C3—C4—H4	118.9	N4—C14—H14	119.0
C6—C5—C10	121.86 (16)	C13—C14—H14	119.0
C6—C5—O1	121.32 (15)	O3—C15—O4	124.21 (19)
C10—C5—O1	116.59 (15)	O3—C15—C7	123.90 (18)
C5—C6—C7	118.28 (15)	O4—C15—C7	111.88 (17)
C5—C6—H6	120.9	O4—C16—H16A	109.5
C7—C6—H6	120.9	O4—C16—H16B	109.5
C6—C7—C8	120.99 (16)	H16A—C16—H16B	109.5
C6—C7—C15	121.16 (15)	O4—C16—H16C	109.5
C8—C7—C15	117.83 (16)	H16A—C16—H16C	109.5
C9—C8—C7	118.26 (16)	H16B—C16—H16C	109.5
C9—C8—H8	120.9	C2—N1—C3	115.66 (17)
C7—C8—H8	120.9	C1—N2—C4	116.18 (17)
C8—C9—C10	122.16 (15)	C12—N3—C13	116.28 (19)
C8—C9—O2	120.22 (16)	C11—N4—C14	115.96 (18)
C10—C9—O2	117.42 (16)	C1—O1—C5	118.77 (13)
C9—C10—C5	118.35 (16)	C11—O2—C9	118.32 (13)
C9—C10—H10	120.8	C15—O4—C16	117.08 (18)
			
N2—C1—C2—N1	−1.4 (3)	C6—C7—C15—O4	−17.0 (3)
O1—C1—C2—N1	−179.07 (16)	C8—C7—C15—O4	164.56 (17)
N2—C1—C2—Cl1	178.52 (14)	C1—C2—N1—C3	−0.5 (3)
O1—C1—C2—Cl1	0.9 (2)	Cl1—C2—N1—C3	179.52 (14)
N1—C3—C4—N2	−0.9 (3)	C4—C3—N1—C2	1.6 (3)
C10—C5—C6—C7	−2.9 (3)	O1—C1—N2—C4	179.72 (16)
O1—C5—C6—C7	171.39 (15)	C2—C1—N2—C4	2.1 (3)
C5—C6—C7—C8	1.1 (3)	C3—C4—N2—C1	−1.0 (3)
C5—C6—C7—C15	−177.37 (16)	C11—C12—N3—C13	−0.8 (3)
C6—C7—C8—C9	1.9 (3)	Cl2—C12—N3—C13	177.75 (19)
C15—C7—C8—C9	−179.61 (16)	C14—C13—N3—C12	0.4 (4)
C7—C8—C9—C10	−3.3 (3)	O2—C11—N4—C14	−179.00 (18)
C7—C8—C9—O2	−178.05 (15)	C12—C11—N4—C14	0.0 (3)
C8—C9—C10—C5	1.5 (3)	C13—C14—N4—C11	−0.4 (3)
O2—C9—C10—C5	176.47 (15)	N2—C1—O1—C5	17.9 (2)
C6—C5—C10—C9	1.6 (3)	C2—C1—O1—C5	−164.46 (15)
O1—C5—C10—C9	−172.90 (15)	C6—C5—O1—C1	59.1 (2)
N4—C11—C12—N3	0.6 (3)	C10—C5—O1—C1	−126.30 (17)
O2—C11—C12—N3	179.63 (18)	N4—C11—O2—C9	0.6 (3)
N4—C11—C12—Cl2	−177.87 (15)	C12—C11—O2—C9	−178.44 (16)
O2—C11—C12—Cl2	1.2 (2)	C8—C9—O2—C11	−78.8 (2)
N3—C13—C14—N4	0.2 (4)	C10—C9—O2—C11	106.15 (18)
C6—C7—C15—O3	163.5 (2)	O3—C15—O4—C16	1.1 (3)
C8—C7—C15—O3	−15.0 (3)	C7—C15—O4—C16	−178.4 (2)

### Hydrogen-bond geometry (Å, º)

**Table:**

*D*—H···*A*	*D*—H	H···*A*	*D*···*A*	*D*—H···*A*
C4—H4···O2^i^	0.93	2.47	3.192 (3)	135

Symmetry code: (i) *x*, *y*−1, *z*.
